# Learning bio-inspired head-centric representations of 3D shapes in an active fixation setting

**DOI:** 10.3389/frobt.2022.994284

**Published:** 2022-10-18

**Authors:** Katerina Kalou, Giulia Sedda, Agostino Gibaldi, Silvio P. Sabatini

**Affiliations:** ^1^ Department of Informatics, Bioengineering, Robotics and Systems Engineering, University of Genoa, Genoa, Italy; ^2^ University of California Berkeley, School of Optometry, Berkeley, CA, United States

**Keywords:** stereopsis, vergent geometry, binocular disparity, active fixations, 3D shape perception, recurrent hierarchical networks

## Abstract

When exploring the surrounding environment with the eyes, humans and primates need to interpret three-dimensional (3D) shapes in a fast and invariant way, exploiting a highly variant and gaze-dependent visual information. Since they have front-facing eyes, binocular disparity is a prominent cue for depth perception. Specifically, it serves as computational substrate for two ground mechanisms of binocular active vision: stereopsis and binocular coordination. To this aim, disparity information, which is expressed in a retinotopic reference frame, is combined along the visual cortical pathways with gaze information and transformed in a head-centric reference frame. Despite the importance of this mechanism, the underlying neural substrates still remain widely unknown. In this work, we investigate the capabilities of the human visual system to interpret the 3D scene exploiting disparity and gaze information. In a psychophysical experiment, human subjects were asked to judge the depth orientation of a planar surface either while fixating a target point or while freely exploring the surface. Moreover, we used the same stimuli to train a recurrent neural network to exploit the responses of a modelled population of cortical (V1) cells to interpret the 3D scene layout. The results for both human performance and from the model network show that integrating disparity information across gaze directions is crucial for a reliable and invariant interpretation of the 3D geometry of the scene.

## 1 Introduction

Three-dimensional shape perception from binocular stereopsis is a common perceptual process employed by animals characterized by forward pointing eyes, such as humans and other primates, for understanding and interacting with the environment. For such a task, our visual system relies on binocular disparity information, as the relative displacement of corresponding image projections of the same object on the left and right retinas (Howard and Rogers, 1995). In humans, the underlying neural mechanisms are already present at only 4 months after birth, together with the ability of perceiving shape from motion (Yonas et al., 1987).

A correct development of binocular vision is mandatory for a reliable perceptual process and for proper coordination of binocular eye movements (Thompson et al., 2015; Milla Baños and Piñero, 2020). The understanding of the three-dimensional (3D) scene is in fact obtained by constantly adjusting gaze and vergence towards the next most salient or informative point for the task at hand (Hinkle and Connor, 2002; Rosenberg et al., 2013). Binocular depth perception is achieved by comparing the left and right retinal images. To do this, we must determine for each point in one image which point in the other originated from the same part of the same object. False matches create the correspondence problem. Solving the stereo correspondence problem is computationally demanding, since a single natural scene has unpredictable complex 3D structure, and retinal corresponding points patterns likely depend on the active fixation strategy, too.

So, how does our visual system successfully recover the 3D shape information using the continuously changing gaze-dependent disparity information provided by an actively fixating binocular system, in a fast and reliable way? Here, we explore the hypothesis that an active fixation geometry is essential for the recognition of an object’s shape in depth. Assuming a patch-wise linear model of the depth structure, a full reconstruction of the scene is not always necessary and the actual binocular geometry of a 3D fixating observer allows our visual system to actively measure only deviations of the internal model’s predictions. In the present paper, our goal is two-fold; 1) to model a plausible cortical pathway of 3D shapes perception through the hierarchical processing of distributed (i.e., population-based) representation of binocular disparity and their corresponding elementary differential components, and 2) to investigate the integration mechanisms of the binocular signal across multiple gaze cyclopean directions in an active fixation setup. In the context of computational systems with limited resources a strategy that limits the number of exploratory eye movements (i.e., saccades) represents an important desirable asset. Towards that goal, we combined the findings from a psychophysical experiment with the outcomes of a trained bio-inspired hierarchical network - where both our human participants and the computational network were asked to classify the orientation in depth of a dataset of planar 3D surfaces. Our participants’ performance together with the learned intra-gaze recurrent weights and the resulting activation patterns of the trained network’s units demonstrate the emergence of reliable gaze-invariant 3D representation.

The rest of the paper is organized as follows: in Section 2, we introduce the problem of recovering the 3D layout of an object or a scene in the case of an active fixating observer, together with a specific experimental investigation on human subjects. In particular, we present the outcomes of a dedicated experiment where participants judged the orientation in depth of a 3D planar stimulus while controlling their active fixation behavior. These behavioral results are compared with those achieved by a trained recurrent hierarchical network described in Section 3. Concluding remarks and a general discussion are presented in Section 4.

## 2 Materials and methods

### 2.1 Structure-from-stereo for an active fixating observers

Recovering the 3D layout of an object or a scene from images is a well formalized problem (Trucco and Verri, 1998). When we are provided with a sufficiently dense disparity information, it enables 3D shape recognition (Marr, 1982; Poggio et al., 1985; Nalpantidis et al., 2008; Li et al., 2009) and classification (LeCun et al., 2004; Fei-Fei et al., 2007). A standard Computer Vision approach relies on a pair of cameras with parallel optical axes, yielding to binocular disparities along the horizontal epipolar lines. This is not the case for natural binocular vision systems, where the stereo images are acquired by pairs of eyes that are in vergent geometry, and that continuously explore the scene by moving the fixation point around the 3D environment (Gibaldi et al., 2017a; Canessa et al., 2017).

A vergent stereo imaging geometry is a powerful means for focusing the attention of a vision system on a particular region of interest. However, the price to be paid is a more complex geometric relationship between binocular corresponding points, especially during visual exploration of the peripersonal space where large values of vergence occur (Sprague et al., 2015; Gibaldi et al., 2021; Aizenman et al., 2022). The zero-disparity condition at fixation, granted by vergence movements, directly influences the pattern of retinal disparity used for estimating the 3D position and orientation of the fixated object. Moreover, the vergence posture has an impact on the accuracy of stereopsis, too. Different eye positions can influence the shape of the empirical horopter (Schreiber et al., 2008; Gibaldi and Banks, 2019) and thus the mechanisms of perceptual vision (Howard and Rogers, 1995). As a consequence, the fixation point, i.e. where the system verges, becomes a reference that can be parameterized by the relative orientations of the eyes.

**FIGURE 1:**
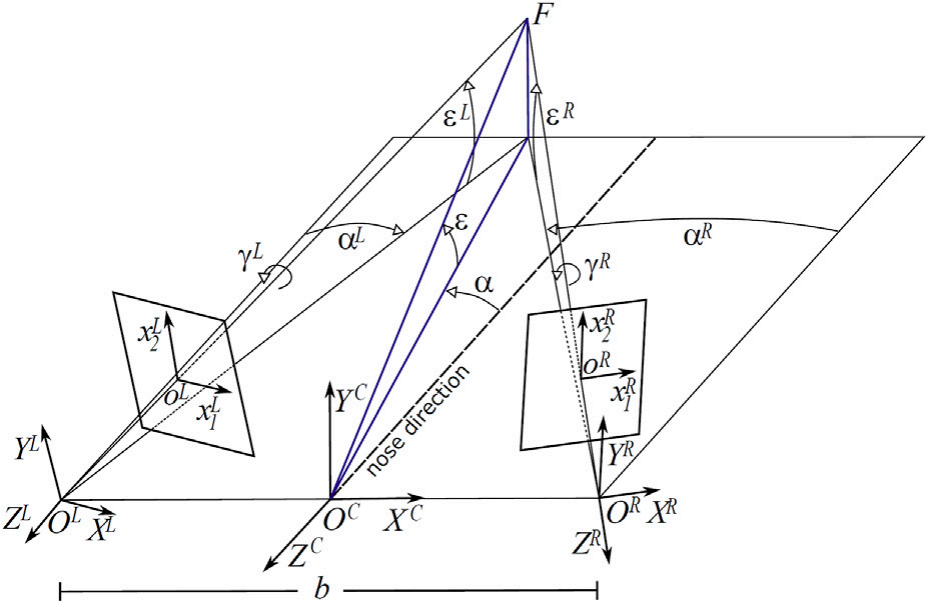
Schematic of the viewing and imaging geometry of a binocular active vision system. *F* is the fixation point, defined by the azimuth *α* and elevation *ϵ* angle pair, *C* is the cyclopic position (halfway between the eyes), *L* and *R* are the left and right camera positions, separated by a baseline *b* = 60 mm. The *ϵ* ^*L*/*R*^, *α* ^*L*/*R*^ and *γ* ^*L*/*R*^ values stand for the elevation (pitch), azimuth (yaw) and torsion (roll) angles of the left L and right R eye.

**FIGURE 2:**
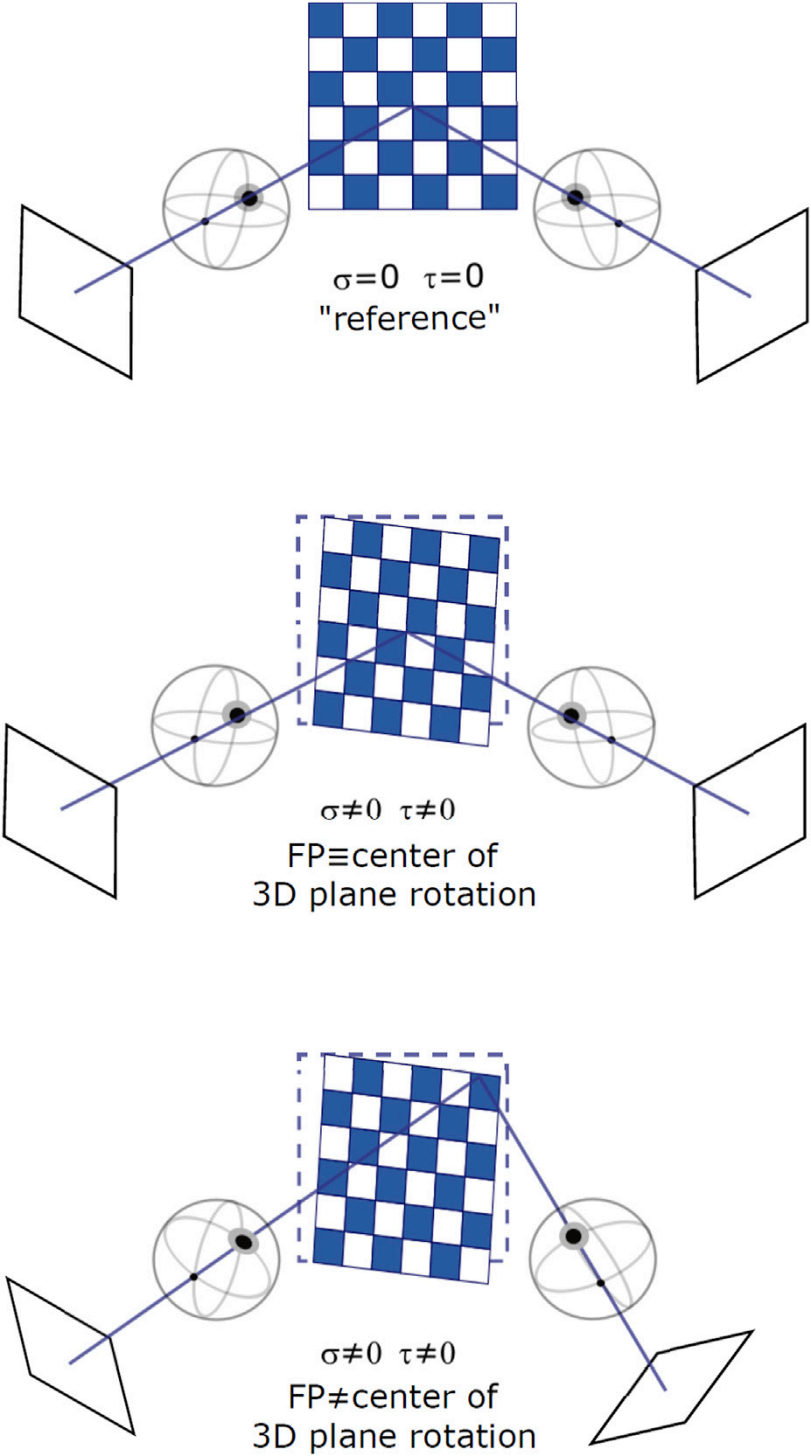
Different binocular viewing conditions for varying stimulus orientation and gaze direction: a frontoparallel plane with straight-ahead gaze (top), a slanted plane with straight-ahead gaze (middle), and a slanted plane with eccentric gaze (bottom). Slant and tilt of the plane are expressed as rotations around a fixed [*X*, *Y*, *Z*] world reference frame.

A convenient way of expressing the binocular posture is by considering azimuth and elevation rotations of the left and right cameras, separated by a baseline *b* = 60 mm, with respect to their straight-ahead (primary) positions. Figure 1 shows the sketch of an active binocular system. Eye position is expressed in a head-centric reference frame. The nose direction is the line orthogonal to the baseline and lying in a transverse plane passing through the eyes. Gaze direction defines the fixation line, the fixation point is at the intersection of these lines on the first visible surface. Since our aim is to simulate the actual images projecting on the retinas of a verging binocular vision system, we generate the stereo pairs through the toe-in method. Each ‘model camera’ is pointed at the target point (the fixation point) through a proper rotation that mimics human eye movements (Gibaldi et al., 2017a; Canessa et al., 2017). Then the left and right views project onto two different planes (see Figure 2). The cameras are characterized by the following parameters (each expressed with respect to the fixed head reference frame): camera position **O** ^*L*/*R*^ and camera orientation $R^{L/R}=R^{L/R}\left(\epsilon^{L/R},\alpha^{L/R}\right)$ , function of the elevation *ϵ* and azimuth *α* angles.

Cameras have pinhole optics with unitary focal length. The origin of the left and the right view volume is fixed at

$$T^{L/R}=\left(\pm \frac{b}{2},0,0\right)$$ (1)

while the cyclopic view volume is located at the origin of the head reference frame. To emulate the behavior of a couple of verging pan-tilt cameras, the complete rotation of each camera is defined by composing in cascade the above rotations following an Helmholtz gimbal system:

$$R^{L/R}=R_{\epsilon }^{L/R}R_{\alpha }^{L/R}.$$ (2)

Considering human binocular eye coordination, the complete 3D pose of the eyes must take into account rotations about gaze directions (i.e., binocular Listing (L2) torsion angles). Accordingly, and compliantly with L2, we pre-multiply the rotation matrices $R^{L/R}$ by a torsional rotation matrix $R_{\gamma }^{L/R}$ to obtain the complete rotation of the view-volumes:

$$R_{L2}^{L/R}=R_{\gamma }^{L/R}R^{L/R}.$$ (3)

For a complete derivation of the *γ* angles refer to (Canessa et al., 2017). In this way, it is possible to insert a camera in the scene (e.g., a perspective camera), to obtain a stereoscopic representation with convergent axes and to decide the location of the fixation point. Thus, for any point in the scene we can obtain its retinal disparity as the difference in retinal position of the left and right projections of the point.

The resulting disparity is defined as a vector ***δ*** = (*δ*_1_, *δ*_2_) comprising horizontal and vertical retinal disparity components (Poggio et al., 1985):

$$\begin{array}{ll} \delta_{1} & =x_{1}^{R}-x_{1}^{L}\\ \delta_{2} & =x_{2}^{R}-x_{2}^{L} \end{array}$$ (4)

measuring the difference of the retinal left $\left(x_{1}^{L},x_{2}^{L}\right)$ and right $\left(x_{1}^{R},x_{2}^{R}\right)$ image coordinates that correspond to the same point in the scene.

**FIGURE 3:**
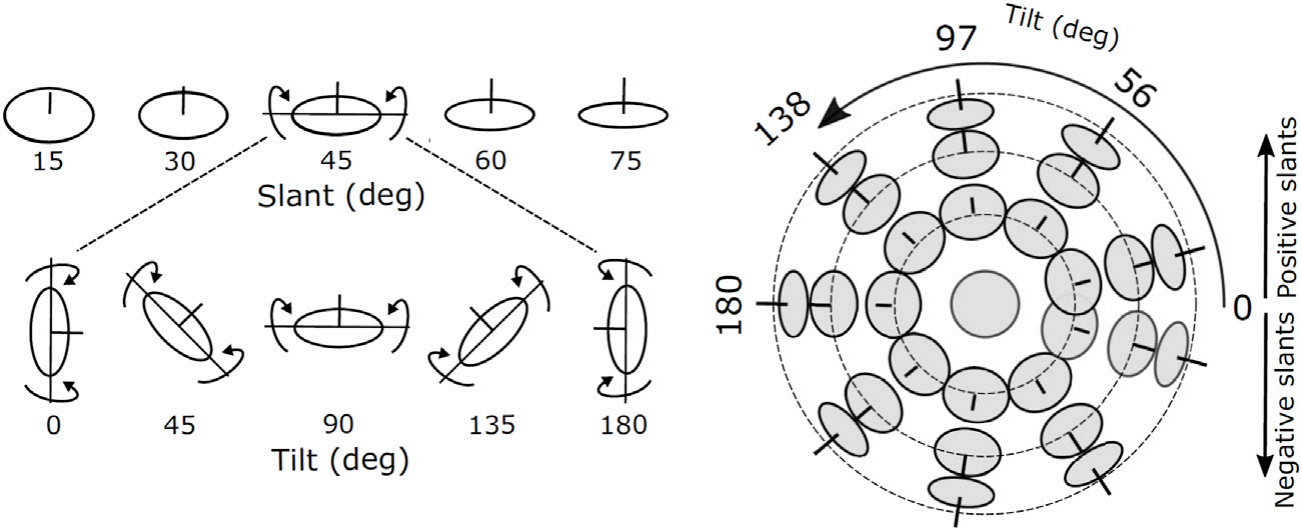
Images of oriented disks showing examples of slants and tilts components of surface orientation. The line segment at the center of each disk is aligned in the direction of the surface normal. The joint slant and tilt components form a spherical coordinate system in which lines of latitude have constant slant, and lines of longitude have constant tilt.

To investigate how the (local) disparity information can be used to estimate the structure of the scene, we consider the simplified problem of estimating the orientation of a planar surface. The orientation of a plane in depth can be parameterized by its two degrees of freedom, namely slant and tilt, defined as successive rotations around a world gravitational reference frame (Stevens, 1983), see Figure 3.

More precisely, the center of rotation of the plane is set at a fixed distance along the intersection of the median plane (vertical in an upright position of the body, perpendicular to the baseline at its center) and the horizontal visual plane (containing the two eyes’ rotation centers); we considered a distance of 350 mm, which is a typical average distance for visually-guided manipulation tasks. Note that as the eye can move over the planar surface, the plane does not necessarily represent the first-order approximation of a smooth physical surface at the fixation point. As a result, the disparity patterns depend both on the viewing geometry and on the surface orientation, making it impossible to recover the disparity pattern from a single measurement (i.e., a single gaze).

Using the ideal observer’s head model described above, we computed the disparity vector field patterns related to differently oriented 3D planes, and for a number of different gaze directions. The 3D plane, as a mesh in space with its center at (0, 0, 350) mm is subtended by a 50°) visual angle. This size was chosen so that it fully remains in the observer’s binocular field of view for every orientation and gaze direction as defined below. As illustrated in Figure 1, the plane’s rotation in depth is defined by composing in cascade two rotations:

$$R=R_{\tau }{\text{R}}_{\sigma }$$ (5)

with *σ*, *τ* being the slant and tilt orientation angles expressed as rotations around the *Y* and *X* axes, respectively, of a fixed left-handed triplet (*X*, *Y*, *Z*) in a world gravitational head-centric reference frame.

The slant and tilt values were sampled on a polar grid where the latitude represents a constant slant angle and the longitude represents a constant tilt angle (Figure 2). This parameterization stems from experimental evidence on the just noticeable differences (JND) of oriented stereoscopic planes in depth (Norman et al., 2006). Indeed, slant-tilt polar encoding naturally decomposes the problem of determining surface orientation into two substantially independent perceptual problems. Psychophysical studies (for example, (Balch et al., 1977)) suggest that both rotation values are encoded as internal visual parameters and that they vary linearly with the objects’ orientation in depth.

**FIGURE 4:**
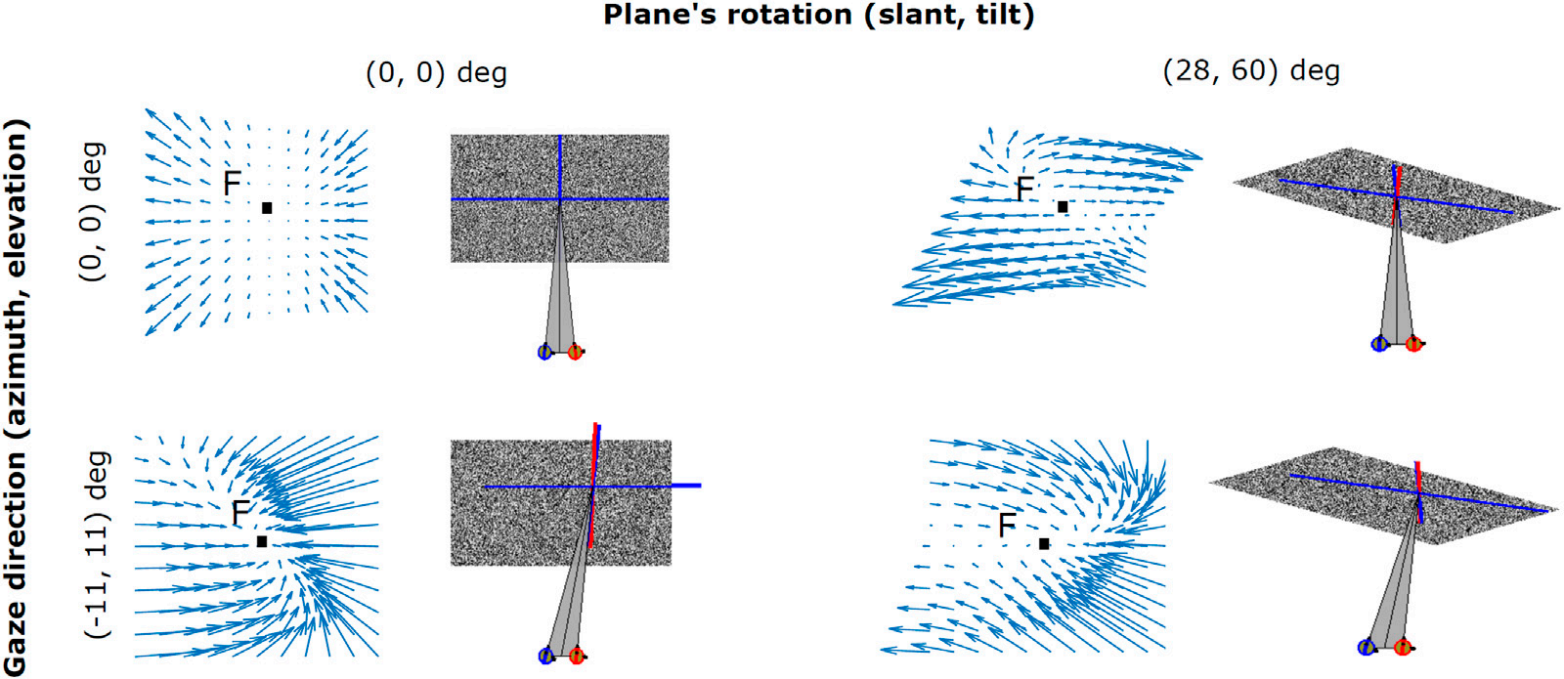
Examples of binocular disparity patterns related to angled planes observed under different viewpoints. Both the change in the cyclopean gaze direction (*α*, *ϵ*) (0°, 0°) and (11°, 11°), and the plane’s rotation in depth (*σ*, *τ*) (0°, 0°) and (28°, 60°) result in distortions (i.e., rotational components) in the disparity vector field.

As shown in Figure 4, the zero-order disparity conveyed for different plane rotations in depth with (*σ*, *τ*), i.e. (0°, 0°), (28°, 60°) and (28°, 300°), provide ambiguous information for recovering the invariant 3D planar orientation. This is due to the fact that, even when the position of the 3D object in space remains stable, the change in the cyclopean gaze direction induces considerably high variance on the disparity signal. An example of this can be seen in the columns of Figure 4), where for the same slant and tilt parameters the disparity information conveyed by our binocular system significantly changes with gaze position. Hence, how does our visual system instantly recover an object’s 3D planar orientation, in a gaze invariant headcentric coordinate system, from a so highly variable disparity signal? Here, we explore the idea that active fixations are a crucial mechanism for the integration of the disparity signal towards a complete understanding of the object’s 3D shape and also, that a single disparity map is able to provide enough information on the plane’s 3D characteristics by making use of its, partially invariant, first-order disparity components expressed as first-order differentials of the disparity vector fields.

### 2.2 The role of eye movements

**FIGURE 5:**
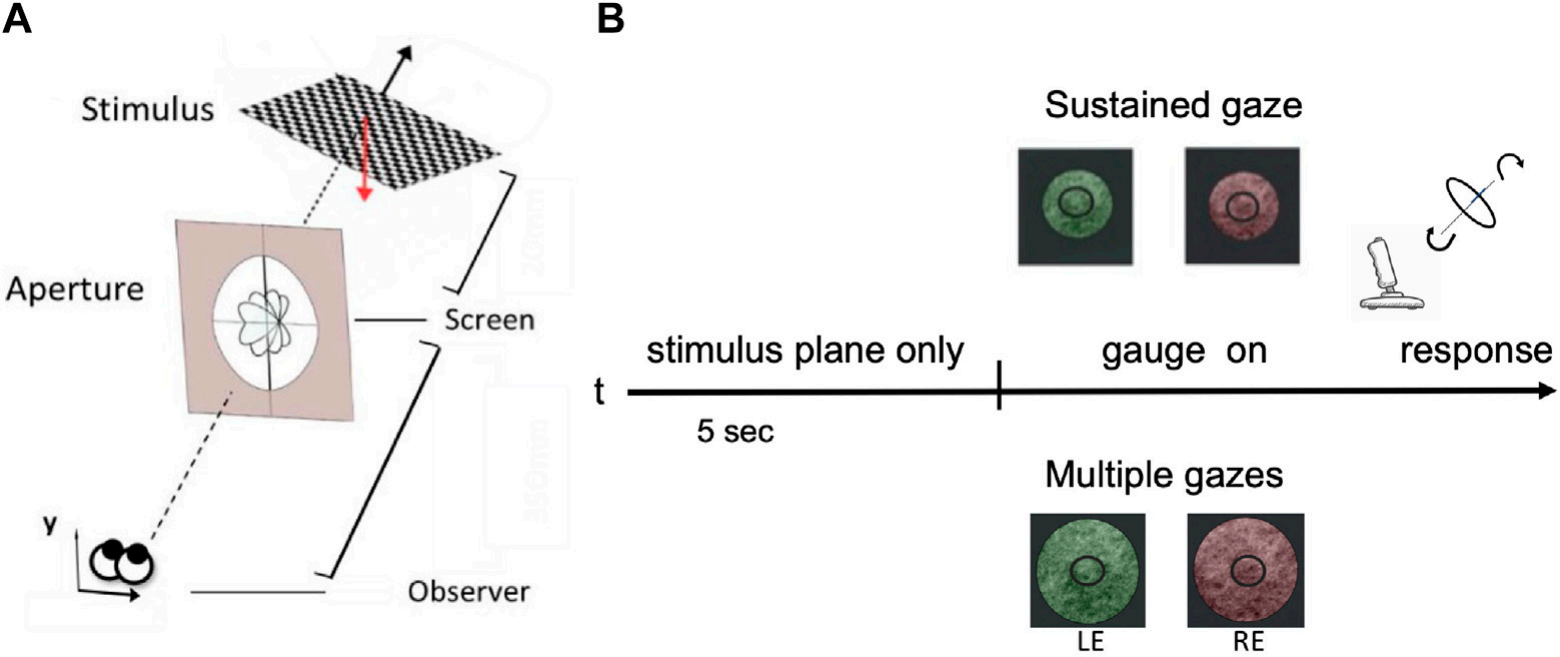
A schematic view of the human slant/tilt perceptual experiment. **(A)** Each 3D oriented plane is rendered stereoscopically. The observer adjusts the orientation of the gauge ring in depth until it appears lying flat on the surface of the planar stimulus. **(B)** The experimental protocol: for the first 5 s participants were presented with a 3D rotated planar surface and were asked to either keep their gaze fixed on a central dot - ‘SG condition’ - or actively explore the stimulus - ‘MG condition’. The two conditions were also parameterized by the aperture size: small one (121 mm of diameter) for the SG and a wider one (242 mm of diameter) for the MG condition. Each stimulus is presented dichoptically in the left eye (LE) and in the right eye (RE).

During the last decades, psychophysical research on structure-from-stereo perception evolved along two main experimental paradigms: simple 3D shapes rotated in depth either by slant or tilt, only (Backus et al., 2001), (Tsao et al., 2003) or complex 3D structures (Yamane et al., 2008), (Georgieva et al., 2009) (Rosenberg et al., 2013). In the first case, researchers usually study the subjective perception of the ordinal or interval differences of 3D surface orientation in depth (e.g Reichel et al., 1995), (Norman et al., 2006), whereas in the second case they are mostly concerned with the more complex underpinnings of 3D shape perception (Koenderink et al., 1992), (Koenderink et al., 1996). For the purpose of the current study, the former approach is too simple and the latter one is too complex. Furthermore, to the best of our knowledge, none of these studies explicitly account for the active fixation condition in a vergent geometry binocular setup. On this ground, we designed a visual psychophysical experiment that employs a variation of the gauge figure technique (Koenderink et al., 1992), (Todd et al., 1997), where participants are asked to judge the orientation in depth of a plane by adjusting a gauge figure superimposed on its surface, while explicitly controlling the joint slant and tilt rotation of each plane, as well as the participant’s active fixation behavior; see Figure 5A. This setup aims to replicate as closely as possible the circumstances of a person that quickly explores and recognizes the orientation of a small object’s hold in her hands.

*Experimental protocol* - As shown in Figure 5B, each stereo image pair was presented on an LG 42LW450A stereoscopic LCD screen (1920 × 1,080 pixels resolution) and viewed dichoptically through a pair of passive polarized glasses. Each 3D planar surface used as a stimulus is rotated in space by one of 81 pre-defined pairs of slant and tilt angles: nine values of slant in the range [4°, 48°] by steps of 4°, and nine values of tilt in the range [30°, 360°] by steps of 4.5°. This parameterization stems from psychophysical evidence on the just noticeable differences (JNDs) of oriented stereoscopic planes in depth ((Stevens, 1983), (Norman et al., 2006)). These orientation pairs were further grouped into nine overall orientation classes for the purpose of comparing our human participants performance with the one of our modelled network described in the subsequent section. This way of parameterization results in a common chance level of 1/9 across our behavioral experiments’ and our computational models’ results.

Participants were seated at a fixed distance of 570 mm from the screen and their heads were restrained by a chin rest. A perceptual matching task was performed. For each trial, participants were presented with a rendered planar surface (test stimulus) for 5 s. After this time window, a gauge ring appeared in a frontoparallel arrangement. Participants were asked to use a joystick (Thrustmaster T16000M FCS) to place the gauge figure so as to appear lying flat on the test stimulus. The joystick’s movement was continuously mapped in to the gauge’s figure slant and tilt angles parameterized as the longitude and the latitude of a spherical coordinate system, respectively. At the moment that the gauge ring was perceived aligned with the planar surface orientation in a satisfactory way, participants were instructed to press the keyboard’s spacebar for ending the trial. For each trial, we measured reaction time and accuracy as to whether the chosen gauge figure’s orientation class matched the one of the presented planar surface underneath it. Subjects had any time limit to provide the answer and the average response time typically decreases over trials.

We explicitly conditioned the participants’ binocular fixation control by introducing two experimental conditions: sustained gaze fixation (SG) and multiple gaze shifts (MG). For the first condition, participants were instructed to keep their eyes strictly on the central fixation dot whereas for the second condition they were encouraged to actively explore the 3D rendered oriented planar object. Participant’s cyclopean gaze direction movements were recorded for both SG and MG conditions, during the initial 5 s period when the participants - actively or not - were invited to perceive the orientation in depth of the test stimulus.

*Subjects* - Behavioral perceptual and motor data were collected from six participants (3 females) with an average age of 35 years with normal or corrected-to-normal binocular vision. All participants have given informed consent for their involvement in this study. The procedure was in accordance with the Declaration of Helsinki. Each participant performed a total of 567 trials (7 repetitions for each of the 81 slant and tilt pairs) for each of the two experimental conditions, sustained gaze fixation and multiple gaze fixations, referred as SG and MG in the following. Each participant randomly started with SG or MG condition, which were equally distributed.

*Apparatus and stimuli* - The 3D geometry of the scene was implemented with the use of the C++/OpenGL architecture extension for Psychophysics Toolbox (Brainard, 1997) for MATLAB (Mathworks) based on the biological principles of cyclopean binocular geometry as described by Hansard and Horaud (2008). More precisely, the stimuli were 3D planar surfaces, simulated as a mesh around a point **P**_0_ = (0, 0, 370) mm, expressed in headcentric coordinates. Each 3D plane was initially defined as an union of all the points **P**_*i*_ that satisfy equation **P** = {**P**_*i*_: **n** ^*T*^ (**P**_*i*_ − **P**_0_) = 0} with **n** being the normal vector of the plane. After that, a gauge figure was initialized as a small circular gray ring with an internal diameter of 6 mm and an external radius of 16 mm to that it covers ≈16 deg of visual field. Its world gravitational center was defined at *P*_*a*_ = (0, 0, 350) mm, coinciding with the center of the screen (cf. Figure 5A). For the subsequent rendering of the 3D shape we followed the experimental paradigm proposed by Gibaldi et al. (2017a) and we used the vergent geometry setup therein defined for the simulation of the ideal observer’s eyes. This setup allows us to consider the disparity cue as a vector field composed of both horizontal and vertical components. More in detail, we initialized the two virtual cameras in space with a baseline *b* = 60 mm parallel to the *X* axis. Each camera was subsequently rotated according to the Helmholtz reference frame, so that its optical axis will pass through the world gravitational point **P**_*a*_ = (0, 0, 350) mm, coinciding with the center of rotation of the gauge figure.

*Eye tracking* - A Tobii EyeX table eye tracker was used, with a frame rate of 60Hz placed at a distance of ≃ 300 mm from the participant’s head. Before the experiment starts, the eyetracker was calibrated for each eye, separately. The recording of the eye movements as well as the calibration procedure relied upon the integrated functions of the Tobii EyeX toolkit for Matlab (Gibaldi et al., 2017b). During each trial, the participant’s current cyclopean gaze direction was computed as the middle point between the left and right 2D gaze points recorded by the eyetracker and approximated to a point of a regular grid at the surface of the screen. For each trial, the 2D fixation points on the cyclopean image of the 3D planar surface were obtained from each cyclopean gaze scanpath by considering a minimum fixation time of 0.3 s and then backprojected and analyzed on a normalized grid superimposed onto the surface of each 3D planar surface. In the SG condition, subjects who had not kept fixation close to the center of the screen (i.e., <0.5 visual deg) were discarded.

**FIGURE 6:**
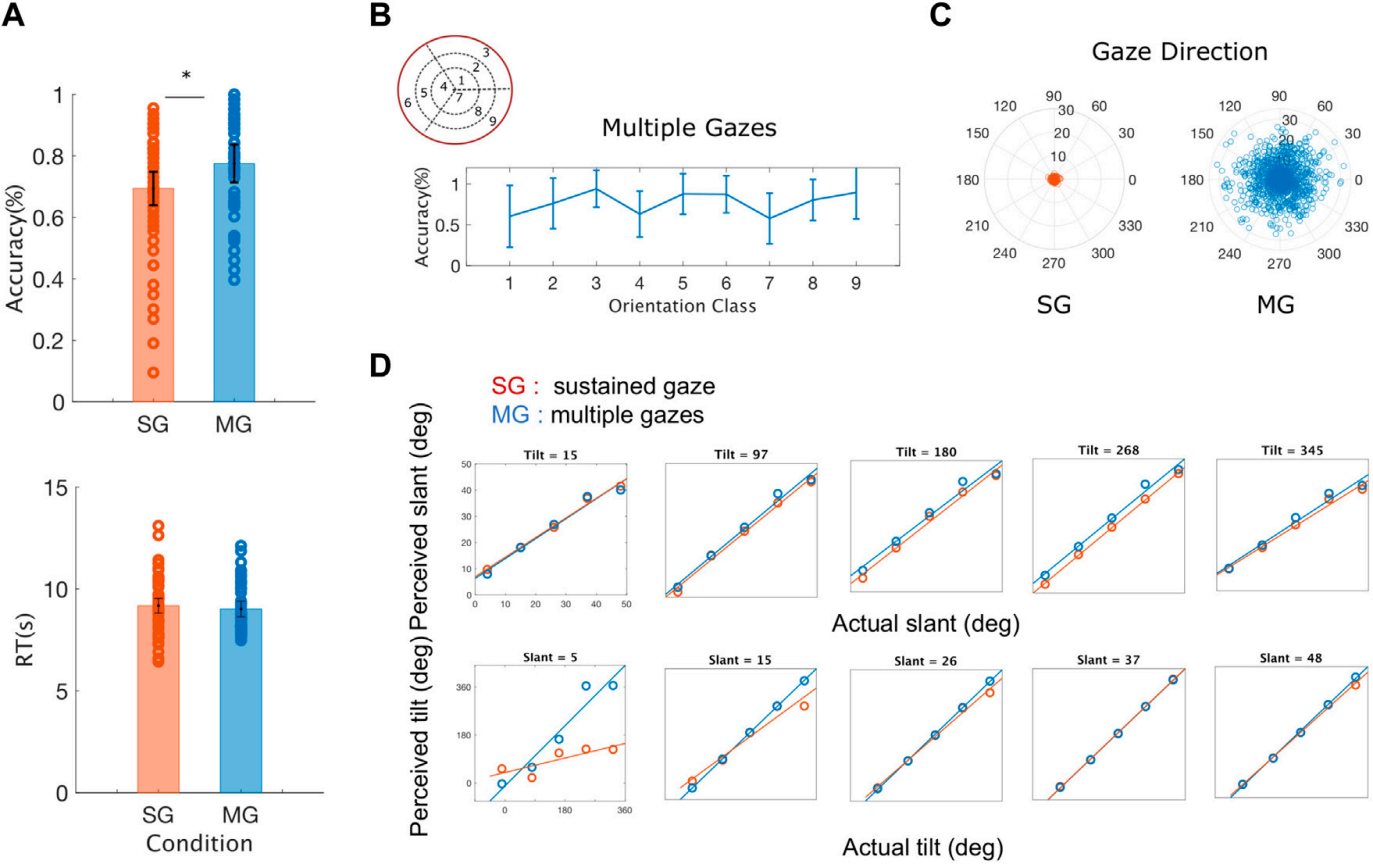
Averaged behavioral results across all the rotation pairs (slant and tilt) and all the participants **(A)** Average accuracy and average reaction time (RT) for sustained gaze fixation (SG) and multiple gaze (MG) conditions. Each circle represents each participant’s accuracy for each of the nine orientation classes and the black lines denote the standard error of the mean (SEM) **(B)** Average accuracy for the Multiple Gazes condition for each of the nine orientation classes. The blue lines denote the standard error of the mean (SEM) **(C)** Gaze directions across all participants and orientation classes plotted for the Single Gaze condition (left) and the Multiple Gaze condition (right). The polar plot’s angle denotes the gaze direction’s orientation whereas its radius represents the gaze direction’s distance from the center of the screen **(D)** Estimated slant and tilt orientation values as a function of the actual plane’s slant and tilt. During the sustained gaze condition (SG) and for small values of slant, participants tend to grossly misjudge the tilt values of the stimulus plane 3D orientation.

*Resulting evidence* - During the MG condition, participants’ accuracy was significantly higher compared to the SG condition, across all slant and tilt combinations (t (5) = -3.3534, p = 0.0203) (Figure 6A). Reaction times were similar between the two experimental conditions, with the reaction times of the SG condition being slightly longer than those measured in the MG condition. A thorough examination of the interactions between the orientation classes in the slant and tilt parametric space and the participant’s accuracy was not possible due to the low number of participants in our study. However, we visually inspected the average accuracy level across all the participants in the Multiple Gaze condition. As we shown in Figure 6B there is a high inter-subject variability in performance - as denoted by the vertical lines representing the Standard Error of the Mean between our participants - across all classes of slant and tilt 3D orientation parametric space. The orientation classes with the largest values of slant across all values of tilt ({3, 6, 9}) appeared to have the highest classification rate and the lowest variance.

The active fixation behavior of our participants varied significantly between the two conditions (SG and MG) with their eye movements covering a smaller distance from the center of the screen during the SG condition (t (5) = 6.5872, p = 0.001) as it can be seen in Figure 6C. The number of gaze directions was similar for both our conditions (≈43) . The average performance level of our participants varied significantly with gaze number across both our experimental conditions as determined by a two-way ANOVA F (2,5) = 31,893, p = 0).

Furthermore, as it can be seen in Figure 6D, there is no significant difference between the experimental condition (sustained vs. multiple gazes) and the estimation of slant across all values of tilt (t (5) = 0.6363, p = 0.5525) whereas the values of the slopes of the best fit lines of perceived to actual tilt were significantly different across all values of slant (t (5) = -3.9109, p = 0.0113). These results agree with previous findings on the amount of slant-induced bias found in the perception of tilt in a natural stereoscopic images, and on the influence on the estimates of the intrinsic cardinal bias in the tilt prior probability distribution (Burge et al., 2016).

Taken together, these results indicate that using an active binocular fixation strategy is more effective not only for a correct perception of the environment’s 3D structure but also for its computational efficiency. In the following section we explore this hypothesis further by training a recurrent hierarchical network to recognise the orientation in depth of the same 3D planar stimuli as the ones used in our behavioral experiment, using as an input the activation of a modeled population of V1 cells collected through a series of different gaze directions.

### 2.3 Biologically-inspired representation of slant and tilt from binocular disparity patterns in vergent geometry

As mentioned before, zero-order disparity information is highly variant as a function of the plane’s rotation, as well as of the binocular fixation point. However, inspired by previous work on optic flow research (e.g. Koenderink and van Doorn, 1979), (Koenderink and van Doorn, 1979), (Verri et al., 1992), it is possible to exploit linear variations of the disparity field to obtain structure-from-stereo information that is invariant to the direction of gaze. These first-order differentials of the disparity field are indeed invariant to the absolute distance of the object with respect to the cyclopean point of view (depth), even though they actually need information on the geometry of the fixation system for recovering the local surface properties, explicitly.

Formally, around any image point (**x**_0_), the disparity field ***δ*** (**x**) can be described as linear deformations by a first-order Taylor decomposition:

$$\delta \left(x\right)=\delta \left(x_{0}\right)+\left(J\delta \right)\left(x_{0}\right)x+higher\;order\;terms$$ (6)

where **x** = (*x*_1_, *x*_2_) is the image point and

$$\left(J\delta \right)\left(x\right)=\left(\frac{\partial \delta \left(x\right)}{\partial x_{1}},\frac{\partial \delta \left(x\right)}{\partial x_{2}}\right)=\left(\begin{array}{c} \nabla^{T}\delta_{1}\left(x\right)\\ \nabla^{T}\delta_{2}\left(x\right) \end{array}\right)$$ (7)

**FIGURE 7:**
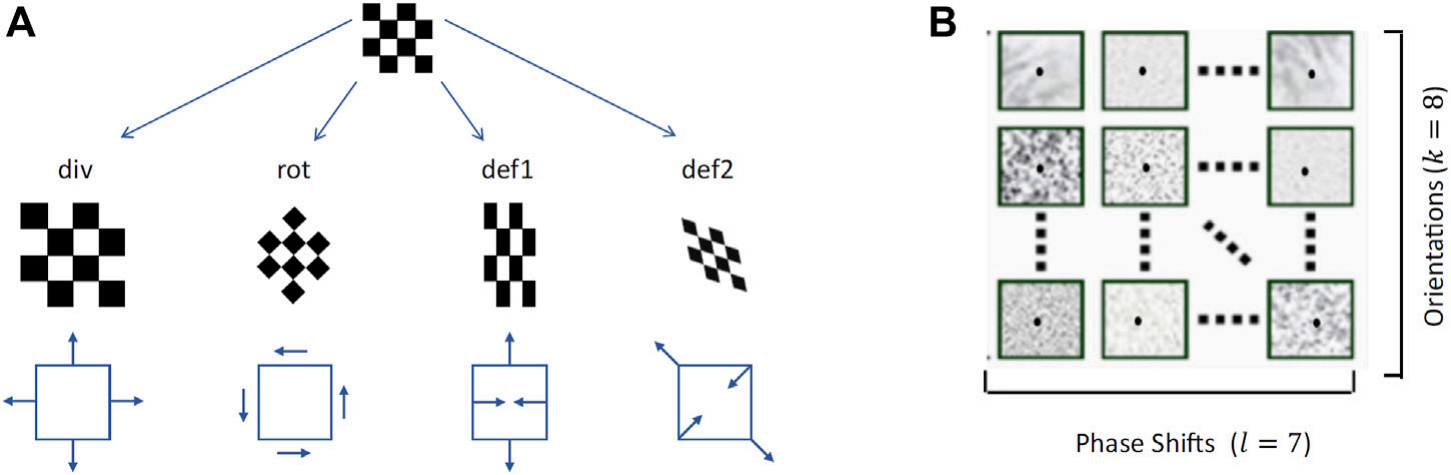
**(A)**The differential elementary components of a vector field, namely divergence, rotation and two components of deformations (Koenderink and van Doorn, 1979) **(B)** Examples of the bio-inspired activation maps of the population of complex binocular neurons (57 maps), for each single slant and tilt rotation pair and each single gaze direction.

By combining the first-order differential components of the disparity field we can obtain its elementary transformations, namely a pure expansion (div), a pure rotation (rot) and two components of deformations (def1, shear and def2, stretching) (Figure 7A):

$$\begin{array}{cccc} div\;\delta \left(x\right) & =\frac{\partial \delta_{1}}{\partial x_{1}}+\frac{\partial \delta_{2}}{\partial x_{2}} & rot\;\delta \left(x\right) & =\frac{\partial \delta_{2}}{\partial x_{1}}-\frac{\partial \delta_{1}}{\partial x_{2}}\\ def_{1}\;\delta \left(x\right) & =\frac{\partial \delta_{2}}{\partial x_{1}}+\frac{\partial \delta_{1}}{\partial x_{2}} & def_{2}\;\delta \left(x\right) & =\frac{\partial \delta_{1}}{\partial x_{1}}-\frac{\partial \delta_{2}}{\partial x_{2}}. \end{array}$$ (8)

**FIGURE 8:**
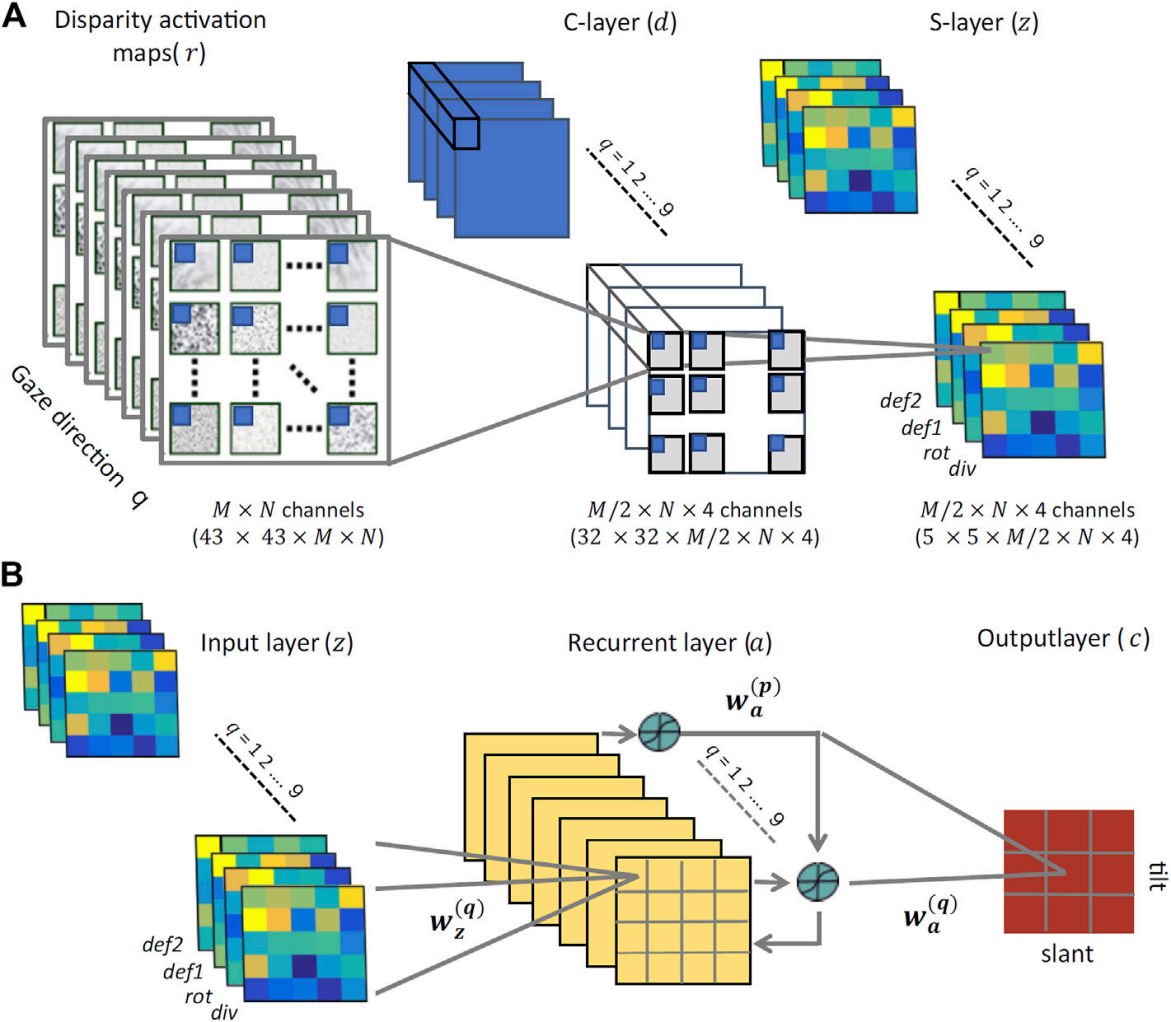
The proposed neural architecture **(A)** The non-trainable preprocessing module is a three-layer convolutional architecture that receives multidimensional disparity-related activation maps for each of the nine gaze directions, and provides a set of neural maps representing disparity differential elementary components (div, rot, def_1_, and def_2_). **(B)** The trainable three-layer module that relies on recurrent connections between its hidden units to learn gaze-invariant disparity representations in the slant/tilt parametric space, from the input neural maps related to a set of different gaze directions.

These four disparity transformations have been found to be quite informative and relatively invariant to the viewing geometry and to the environmental noise, thus an ideal input for a hierarchical network trained to discriminate planar surface orientations, as detailed in the following. On the basis of such differential invariants - and by recurrently combining information associated to the same surface orientation fixated with different gaze directions - we will show that it is possible to gain reliable complex visual descriptors to properly tile the (*σ*, *τ*) space. These descriptors will be obtained by the hard-wired convolutional stage of the network and used as input to the subsequent recurrent trainable stage, as illustrated in Figure 8.

#### 2.3.1 Encoding disparity information from built-in binocular energy detectors

As the front-end of the network architecture that we will propose in the next section, we have a distributed coding of the binocular disparity across different orientation channels through a filtering stage that resembles the filtering process of primary visual cortex (area V1). Disparity information is extracted from a sequence of stereo image pairs by a population of simple and complex cells. Each simple cell response *r*_*s*_ (**x**; Δ*ψ*) is obtained through a linear binocular Gabor-like receptive field *g*_*L*_(**x**) + *g*_*R*_(**x**) positioned in corresponding points **x** of the left and the right images, oriented by the same angle with respect to the horizontal axis, and characterized by the same peak frequency *ω*_0_ and spatial envelope. A proper binocular phase shift (Δ*ψ* = *ψ*_*L*_ − *ψ*_*R*_) between the left and right receptive field along the direction *θ* orthogonal to the orientation of the receptive fields confers to the cell a specific disparity sensitivity along that direction.

According to the binocular energy model (Ohzawa et al., 1990; Qian, 1994; Fleet et al., 1996), the response of a complex cell *r*(**x**) finalizes its tuning by taking the sum of the squared response of a quadrature pair of simple cells:

$$r\left(x\right)=r_{s}^{2}\left(x,\Delta \psi \right)+r_{s}^{2}\left(x,\Delta \psi +\pi /2\right).$$ (9)

Hence, *r*(**x**) has its maximum when the product of the magnitude of the stimulus disparity and the spatial peak frequency equals the phase difference in the binocular receptive field,.

#### 2.3.2 Building distributed representation of first-order disparity field differentials

For each image position, binocular disparity ***δ*** (**x**) can be equivalently defined with respect to any orthogonal coordinate system, rotated by an angle *θ*_*k*_:

$$\delta^{\left(k\right)}\left(x\right)={\left(m_{1}^{\left(k\right)},m_{2}^{\left(k\right)}\right)}^{T}\delta \left(x\right)$$ (10)

where $m_{1}^{\left(k\right)}={\left(\cos\theta_{k},\sin\theta_{k}\right)}^{T}$ and $m_{2}^{\left(k\right)}={\left(-\sin\theta_{k},\cos\theta_{k}\right)}^{T}$ are equivalent generators that point in direction *θ*_*k*_ and $\theta_{k}+\frac{\pi }{2}$ , respectively.

With respect to such a rotated basis, vector disparity differentials can be properly defined through directional derivatives:

$$\left(J\delta^{\left(k\right)}\right)\left(x\right)={\left(m_{1}^{\left(k\right)},m_{2}^{\left(k\right)}\right)}^{T}\left(\begin{array}{c} \nabla^{T}\delta_{1}\left(x\right)\\ \nabla^{T}\delta_{2}\left(x\right) \end{array}\right)={\left(m_{1}^{\left(k\right)},m_{2}^{\left(k\right)}\right)}^{T}\left(J\delta \right)\left(x\right).$$ (11)

Considering that the neuromorphic energy detectors act through different orientation channels, each disparity value, defined in $R^{2}$ can be (redundantly) mapped in the responses **r** of a population of neurons defined in $R^{N}$ , where *N* = *L* × *M*, with *M* is the number of cortical orientation channels, and *L* is the number of the specific phase-based disparity tuning values, for each orientation. The ordered vector space of the *N*-tuples of these responses can be conveniently denoted by two indices: *k* = 1, *…* , *M*/2 that represents the direction *θ*_*k*_ of the disparity component to which the binocular disparity detector is tuned^1^, and *l* = 1, *…* , *L* that represents the specific (scalar) value of tuning Δ*ψ*_*l*_ along direction *θ*_*k*_. Consistently with previous definitions, we can map zero-order disparity ***δ*** ^(*k*)^ (**x**) into ${\left(r_{1,l}^{\left(k\right)}\left(x\right),r_{2,l}^{\left(k\right)}\left(x\right)\right)}_{l=1,\ldots ,L}^{T}$ . Similarly, first-order differentials (**J*δ*** ^(*k*)^) (**x**) map into ${\left(\nabla^{T}r_{1,l}^{\left(k\right)}\left(x\right),\nabla^{T}r_{2,l}^{\left(k\right)}\left(x\right)\right)}_{l=1,\ldots ,L}^{T}$ where $\nabla^{T}r_{i,l}^{\left(k\right)}=\left(\partial r_{i,l}^{\left(k\right)}/\partial x_{1},\partial r_{i,l}^{\left(k\right)}/\partial x_{2}\right)$ .

By composition of dyadic components, we can eventually map the set of disparity differential invariants into the tensor fields of the neural population activity, for each (*k*, *l*) channel:

$$div\;\delta \left(x\right)\mapsto {\left\{\frac{\partial r_{1,l}^{\left(k\right)}}{\partial x_{1}}+\frac{\partial r_{2,l}^{\left(k\right)}}{\partial x_{2}}\right\}}_{\begin{array}{c} k=1,\ldots ,M/2\\ l=1,\ldots ,L \end{array}}$$ (12)

$$rot\;\delta \left(x\right)\mapsto {\left\{\frac{\partial r_{2,l}^{\left(k\right)}}{\partial x_{1}}-\frac{\partial r_{1,l}^{\left(k\right)}}{\partial x_{2}}\right\}}_{\begin{array}{c} k=1,\ldots ,M/2\\ l=1,\ldots ,L \end{array}}$$

$$def_{1}\;\delta \left(x\right)\mapsto {\left\{\frac{\partial r_{2,l}^{\left(k\right)}}{\partial x_{1}}+\frac{\partial r_{1,l}^{\left(k\right)}}{\partial x_{2}}\right\}}_{\begin{array}{c} k=1,\ldots ,M/2\\ l=1,\ldots ,L \end{array}}$$

$$def_{2}\;\delta \left(x\right)\mapsto {\left\{\frac{\partial r_{1,l}^{\left(k\right)}}{\partial x_{1}}-\frac{\partial r_{2,l}^{\left(k\right)}}{\partial x_{2}}\right\}}_{\begin{array}{c} k=1,\ldots ,M/2\\ l=1,\ldots ,L \end{array}}.$$

Note that all derivatives can be approximated as sort of scale-space differentials of the neural population responses:

$$\frac{\partial }{\partial x_{j}}r_{i,l}^{\left(k\right)}≃\frac{\partial }{\partial x_{j}}G_{s}*r_{i,l}^{\left(k\right)}.$$ (13)

Consequently, information about the local differential structure of the disparity map is gained through the computation of the local differential geometry of the neural maps.

## 3 Results

### 3.1 Learning gaze-invariant active 3D shape recognition

The use of a naturalistic stereoscopic experimental setup as described in Section 2.2, gave us the opportunity to investigate how the human visual system integrates multiple, gaze-dependent, pieces of disparity information towards a head-centric invariant representation. From a computational point of view, data provide compelling evidence of a key role of binocular eye coordination in active fixation, for correctly categorizing a global 3D shape property; the planar orientation. However, to date the plausible underlying biological mechanisms responsible for this integration remains rather elusive. In this part of the paper, we tackle this problem from a modeling point of view, by relying on a wide dataset of disparity information of 3D oriented planes obtained by our simulator for training a cortical-like neural architecture to classify planar orientation in depth. The whole multi-layer recurrent network architecture is illustrated in Figure 8.

The proposed architecture comprises two distinct stages: preprocessing and training. The preprocessing stage behaves as a convolutional neural network with two non-trainable layers. The binocular energy input consists of *L* × *M* maps of 43 × 43 pixels. For each orientation (*k*) and phase (*l*) channel, and for each gaze direction (*q*), the activity **d**_*j*_ of unit *j* in the first layer, is given by:

$$d_{j}=W_{\;r}r_{j}$$ (14)

where *W*_r_ is a set of 12 × 12 two-dimensional Gaussian derivative kernels

$$W_{\;r}=\frac{\partial^{m+n}}{\partial x_{1}^{m}\partial x_{2}^{n}}G\left(\;\right),\;m,\;n=0,1\;m+n=1$$ (15)

and **r**_*j*_ is the 12 × 12 binocular energy population response captured by the *j*th unit. This operation yields a set of 4 × *L* × (*M*/2) neural maps of 32 × 32 pixels representing a set of first-order differentials, which are pairwise combined to obtain information about elementary disparity field components (cf. Eq. 8). Each component is eventually pooled by means of a 8 × 8 sliding Gaussian kernel *W*_d_ to obtain the activity of the second convolutional map.

Accordingly, for each orientation and phase channel, and for each gaze direction, the activity **z**_*j*_ of the *j*-unit is given by:

$$z_{j}=W_{\;d}d_{j}$$ (16)

where **d**_*j*_ is the 8 × 8 input from the previous layer captured by the *j*th unit. This process results in a set of 4 × *L* × (*M*/2) neural maps discretized into 5 × 5 pixels, that conveys, for each gaze direction, information about (slant, tilt) orientation pairs. The dataset is finally normalized to have zero mean and [ − 1, 1] magnitude range, and divided in 70% and 30% for the training and test sets, respectively.

The training stage consists of a three-layered network that we will train with a supervised learning algorithm. To each input pixel it corresponds a **z**_*j*_ unit. The output units encode the slant and tilt of the oriented surface. The hidden units receive contributions from the input units and from other hidden units, and relay their output activation to the output units. The input layer consists of nine ‘gaze blocks’ trained in a batch mode. Connections from the input layer to each hidden unit are initialized according to a radially symmetric 4 × 4 Gaussian profile that gradually decreases with the distance between each input unit *i* and the corresponding hidden layer unit *j*. The whole stack of gaze-dependent neural maps **z** feed-forwardly projects to a corresponding single hidden layer, resulting in a four-to-one reduction in dimensionality.

Hidden layers include recurrence between all gaze-related hidden units, which ensure the exchange of information and thus the emergence of gaze-invariant 3D shape descriptors. More specifically, each hidden unit *j* receives an input from the activation **a**_*j*_ of all the units belonging to the same hidden layer map (i.e., gaze direction *q*) as well as inter-maps contributions from the units of the maps related to all the other gaze directions. As a whole, the activation of each hidden layer unit *j* can be written as:

$$a_{j}^{\left(q\right)}=S\left(\underset{\text{ FF}-\text{term}}{\underset{︸}{w_{\;z}^{\left(q\right)}z_{j}^{\left(q\right)}}}\;\;+\;\;\underset{\text{ recurrence}}{\underset{︸}{\underset{p}{\sum }w_{\;a}^{\left(p\right)}a_{j}^{\left(p\right)}}}\;\;+\;\;b_{j}^{\left(q\right)}\right)$$ (17)

where *S* (⋅) is a sigmoid function and **b**_*j*_ the activation bias. The input *W*_*z*_ and the recurrent weights *W*_*a*_ are initialized Gaussian kernels introducing smooth decreasing functions between the two spatial dimensions of the input and the respective hidden layer units. Finally, the weighted output of all hidden units, as shown in Figure 8B, linearly project to a set of nine output classes **c**_*j*_ that encode univocal 3D surface orientations (slant and tilt) in head-centric coordinates:

$$c_{j}=\underset{q}{\sum }w_{\;a}^{\left(q\right)}a_{j}^{\left(q\right)}.$$ (18)

The connection weights between each hidden layer unit and each output unit are initialized as Gaussian kernels of 3 × 3 pixel resolution and standard deviation equal to 1.5 pixel.

A modified version of the Backpropagation Through Time algorithm (BTT) was used to operate in batch mode as in Liu and Van Hulle (1998). The BTT algorithm considers a special case of the general gradient descent backpropagation algorithm Rumelhart et al. (1986), where the weights are updated through a number of steps defined by the number of recurrent connections between their layers. For a given 3D orientation category, the desired output was 1 for the corresponding output unit and 0 for all other units (1-out-of-N coding). More in detail, at each iteration of the learning algorithm, the weights were updated as $w_{\;z}^{\left(q\right)}\leftarrow w_{\;z}^{\left(q\right)}-\lambda \partial E/\partial w_{\;z}^{\left(q\right)}$ , $w_{\;a}^{\left(p\right)}\leftarrow w_{\;a}^{\left(p\right)}-\lambda \partial E/\partial w_{\;a}^{\left(p\right)}$ , $w_{\;a}^{\left(q\right)}\leftarrow w_{\;a}^{\left(q\right)}-\lambda \partial E/\partial w_{\;a}^{\left(q\right)}$ , until the logistic error *E* was below 0.1. Note that the partial derivatives in the weight update equations measure the rate of increase of *E* with respect to the changes in different dimensions of $w_{\;z}^{\left(q\right)}$ , $w_{\;a}^{\left(p\right)}$ and $w_{\;a}^{\left(q\right)}$ .

#### 3.1.1 Network implementation and simulation details

After simulation of an actively fixating binocular head as described in Section 2.1, we built the input to our network, corresponding to 144 unique sampling points of slant and tilt angles: 12 values of slant in the range [4°, 48°] by steps of 4.8°, and 12 values of tilt in the range [30°, 360°] by steps of 30°. The ranges for the slant and tilt values were the same as those used in the psychophysical experiment described above, whereas the sampling frequency was increased to provide us with a richer input for training the network. Subsequently, these slant and tilt combinations were categorized into nine overall planar orientation groups to serve as network’s classification outputs. For each of the original 144 planar orientations, the initial cyclopean gaze direction was defined by the initial pair of azimuth and elevation angles [*α*_0_, *ϵ*_0_] = [0, 0] coinciding with the plane’s rotation center. Following that, we sampled each new gaze direction pair as ${\vec{u}}_{L}=\left(\alpha_{0}+\Delta \alpha ,\;\epsilon_{0}+\Delta \epsilon \right)$ where Δ*α* = Δ*ϵ* = { − 5°, 0°, 5°} resulting in a 3 × 3 gaze grid, for each oriented surface. This results in an overall dataset of 144 × 9 stereo image pairs, serving as the input of the convolutional neural network. Each image has a 123 × 123 pixel resolution, which, for the adopted focal length of 350 mm and linear size of the image sensor of 210mm, subtends a visual field of ≃ 20 ° × 20 °.

For each of the so generated stereo image pair, the binocular disparity information was encoded in the distributed activity of the population of binocular energy neurons. The population of binocular energy detectors is composed of 56 cells for each pixel sensitive to as much values of vector disparities, according to 7 magnitude values and along 8 orientations uniformly distributed between 0 and *π*.

#### 3.1.2 Active recognition results

The algorithm’s performance and the activation of its output and hidden (middle layer) units were tested, using a procedure similar to the one used in electrophysiological experiments for single cell recordings. As an input we used the bioinspired population response to 3D surfaces rotated in depth with rotation values of slant and tilt belonging to the same 12 × 12 dataset - excluding those used for the training procedure (cutoff rate 70% training and 30% test values). The training algorithm was run for 500 iterations with a learning rate *λ* = 0.2 until the logistic error *E* reached a level of >0.001 . After the end of the training procedure, the proposed hierarchical recurrent network reached a level of 100% accuracy on the training set and of 97% on the test set, over all orientation classes. Since the random selection of the test data excludes any potential bias, the source of the 3% error is possibly due to the entrapment of the cost function in a local minima - a well-known vulnerability of neural networks with long recurrent temporal series (Medsker and Jain, 2001). Another potential limitation concerns the fact that the accuracy of the network appears to be higher for extreme values of slant (independently of the value of tilt). This result suggests the need of a finer sampling of the 3D orientation parametric space for spanning a greater number of output classes, and thus better analysing the inter-class discriminability; this will be addressed in a future work. For testing the robustness of the trained algorithm we de-noised the disparity input with a Gaussian filter of gradually higher Standard Deviation (SD = [0.1 : 80] by steps of 0.7. The resulting differential responses of our modeled cortical cells, for all the nine gaze directions, were processed through the network by using the learned weights. For each of the nine orientation classes, the accuracy level of the corresponding output units was averaged over all slant and tilt pairs that belong to the same class.

**FIGURE 9:**
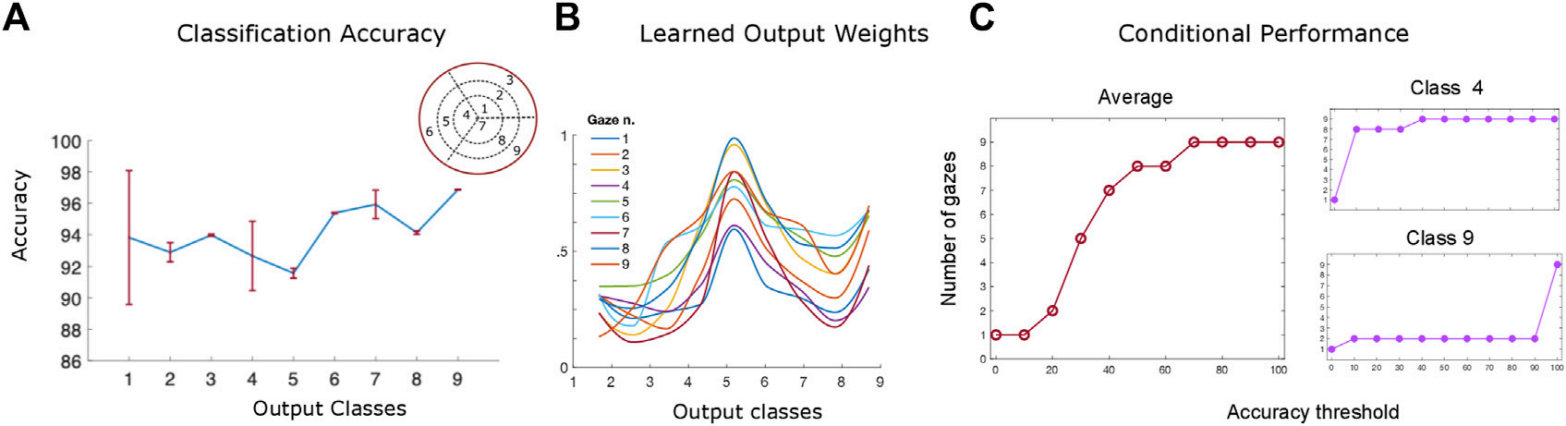
**(A)** The network’s performance (accuracy) averaged over all slant and tilt pairs belonging to each of the nine orientation classes. The red lines denote the standard error of the mean (SEM) across all different slant and tilt pairs belonging to each class **(B)** The trained weight parameters between the recurrent and output layers for orientation class 5, showing the emergence of an invariant slant and tilt representation across all nine different gaze directions **(C)** The algorithm’s performance as a function of the number of gaze-variant disparity maps used as an input averaged across classes (left) and for the orientation classes 9 and 4 (right).

As shown in Figure 9A, the trained algorithm’s performance varies between 93% and 97% with no statistically significant differences between the orientation classes. Interestingly, the classes belonging to the inner ({1, 4, 7}) and outer ({3, 6, 9}) orientation ‘rings’ (i.e the ones with the smaller and largest values of slant across all values of tilt) appear to reach higher accuracy levels than the middle ‘ring’ ones (Marr, 1982; Yonas et al., 1987; Hinkle and Connor, 2002). This may be due to the fact that the disparity input maps with extreme values of slant are more easily classified by the algorithm than the middle level ones, suggesting a potential limitation of the network that will be addressed in future work (for example by including a more detailed sampling of the 3D orientation input space). It is also worth noticing that tilt does not appear to affect the classification performance of the algorithm. Furthermore, the algorithm’s classification performance has higher variance when it comes to small values of slant across all levels of tilt classes (Howard and Rogers, 1995; Trucco and Verri, 1998; Thompson et al., 2015) as it can be seen by the standard error of the mean (95% confidence interval) in Figure 9A. Overall these results confirm the robustness of the trained network’s parameters and agree with the performance of our human participants.

Figure 9B, shows an example of trained weights between all the nine recurrent units of the network’s middle layer and the output orientation class 5 neuron (slant from 20 deg to 32 deg by steps of 4 deg, and tilt from 150 deg to 240 deg by steps of 30 deg). The learned bell–shaped profile centered around output unit 5 in the *x* - axis of the plot, permits the transformation from the retinocentric information of the input disparity components to the headcentric information of the orientation of the 3D surface in depth in an absolute, gaze invariant, way. This result is in accordance with the neurophysiological evidence of the functional progression of depth representation in higher visual cortical areas in primates and humans (Orban, 2008). The activations of these units, indeed, appear to be modulated in amplitude under the same tuning constraint, as in the case of gain field neurons (Salinas and Abbott, 1996). This is a valid clue that the trained recurrent activation patterns in response to specific 3D rotated planar stimuli, may be indeed the origin of an abstract representation in our visual cortical pathway, moving from a retino-centric to a gaze invariant coordinate system.

One of the main goals of this paper was to investigate the role that the binocular active movements play in the recognition of an object’s 3D rotation and shape and whether it is possible to model the action-perception cycle in a vergent geometry setup. Towards that goal, we examined the interplay between the algorithm’s performance and the number of gaze - defined disparity maps we use as an input. In other words, we wondered how many ‘saccades’ does our trained hierarchical network need to reach a given accuracy threshold for each different orientation class. The left panel of Figure 9C shows the average gaze-defined disparity inputs needed by the algorithm for reaching increasing accuracy thresholds. That result is directly comparable with our participants’ overall accuracy levels in our psychophysical experiment, previously displayed in Figure 6A.

Furthermore, as it can be seen in Figure 9C (right), while for the orientation class 9 (*σ* ≈ 42° and *τ* ≈ 303°) the algorithm needs only two gaze defined disparity inputs to achieve an accuracy threshold of up to 90%, on the other hand for orientation class 4 (*σ* ≈ 9° and *τ* ≈ 180°) it needs all nine gazes to reach a classification accuracy above chance level. This in accordance with our psychophysical results described in the previous section where, during the sustained gaze condition, our participants’ performance is significantly modulated as a function of the value of slant of the 3D stimulus plane.

## 4 Discussion

As it occurs for human vision system, active strategies, like foveation, are adopted by natural systems to cope with bandwidth limitations of the retinocortical pathway. When we consider stereoscopis vision, the advantage of active fixation becomes even more compelling as it allows a reduction of the search zones within which binocular correspondences have to be found. Unfortunately, eye movements dramatically complicate the geometrical problems implying the motion of epipolar lines, and make visual information highly dependent on the contingent fixation point. As a whole, vision processes become inescapably related to the fixation strategy, which must be selected or learnt by trading off the cost of eye movements for the accuracy of the recognition performance, to eventually obtaining an efficient gaze-invariant 3D shape understanding.

In this paper, we have employed a bio-inspired population of modeled cortical energy neurons, developed by some of the authors (Chessa et al., 2009), (Gibaldi et al., 2010), (Gibaldi et al., 2016), to train a recurrent hierarchical network that uses as input the responses of a population of cortical disparity detectors. We trained the network to classify the binocular input into the relevant combination of slant and tilt planar orientation, invariantly to the current binocular gaze direction. Specifically, a cascade of a feed-forward (FF) and recurrent network is adopted. The first FF network received the outputs of horizontal and vertical disparity detectors and processes them to obtain approximations of disparity field first-order differentials. We incorporated this network into a closed-loop system along which a set of gaze blocks to integrate perception across eye movements. Recurrent interactions between all gazes ensure the exchange of information and eventually the emergence of gaze-invariant 3D descriptors. The dataset of the population’s activation patterns to validate network’s results was collected by using the same 3D planar stimuli adopted in the psychophysical experiment. To this end, an active vision simulator was used, implementing the biological principles of vergent geometry as described by Hansard and Horaud (2008). By analysing the data of the dedicated psychophysical experiment, we observed that subjects were not only significantly more accurate, but also faster to detect the 3D planar object’s orientation when they actively perform free fixation movements on the surface of the plane (multiple gaze condition) than when their gaze was kept fixed in the center. Remarkably, the artificial network “developed” a similar behavior, suggesting that the active integration of the disparity signals across a number of gaze directions is a crucial mechanism of a binocular vision system, towards an active perception of the 3D environment in a head-centric coordinate system.

The learned weights and the activation patterns of our three-layered recurrent architecture agree with evidence on the existence of a short-hierarchy network that involves the mid-stages of the visual cortical pathway. The response profiles of the trained output units closely resemble the tuning functions observed for neurons in the areas V3/V3A and MT (Orban, 2008) and agree with the activation patterns referenced by Salinas and Abbott (1996) on the existence of populations of gain-modulated neurons in the sensorimotor occipito-parietal networks. Taken together, the results provide compelling evidence that it is possible to train a recurrent compositional network to perceive the local 3D orientation of a planar surface in depth from distributed representations of binocular disparity fields and of their elementary differential components, and to integrate this differential disparity information across multiple binocular fixations, thus capitalizing on the active fixation set-up.

It is plausible that the visual system develops convenient visual descriptors of 3D object shape concurrently with the capacity of making convenient exploratory fixations on their surfaces. As a further step in assessing the role of eye movement in our network, its potential for predicting the next most informative gaze direction deserves a discussion. In order to give a flavour of the capacity of the approach, we progressively fed the network gaze blocks with gaze-contingent disparity information one at a time. Accordingly, we randomly selected a gaze direction as the initial condition and we provide the network with the corresponding visual input only, whereas the remaining hidden layers maps receive a null input (representing the lack of activation of their corresponding neuronal population input). Then we measured the accuracy of the output layer, as well as the recurrent activation **a** ^(*q*)^ (*q* = 1 … 9) of the hidden layer maps. The hidden map with the highest activation indicates the gaze block “co-active” with the current gaze during training, for the specific class of the 3D orientation input. Thus, the next gaze direction is identifies and the corresponding new disparity input added to the pool for the next iteration processing. The procedure was repeated for the number of iterations necessary to achieve at least a 75% classification rate. Results for four 3D planar orientation example demonstrated that the network was always capable of achieving the desired classification accuracy after ≃ 3 eye movements. Each fixation scanpath for a given 3D orientation is unique and characteristically distinct from the others, thus indicating that some disparity patterns associated with gazes can be more informative than others to develop reliable slant/tilt detectors. Active fixation is a complex behavior of our visual system that comprises changes in different parameters such as the number of gazes, the amplitude and directions of their shifts with respect to the center, the latency of sequential eye movement, *etc.* The present paper does not claim that solely the number of gaze directions improves human performance in perceiving the 3D shape of objects in the natural environment. Yet, it tries to explore the fact that active fixational behavior is beneficial for our visual perception and as such should be more often included as a valuable part of computational models of image classification, instead of being considered as a hindrance. For that reason, it was not possible for us to make a one to one comparison between the classification performance of our model as a function of the number of gaze defined disparity input and the accuracy of our participants achieved in the two experimental conditions. However, overall, restricting the participant’s active gaze movements resulted in a lower classification accuracy even for very trivial tasks. The classification results of our algorithm suggest that this could be due to the lower variability of disparity information conveyed to their visual system.

## Data Availability

The raw data supporting the conclusions of this article will be made available by the authors, without undue reservation.

## Footnotes

1 By considering *M*/2 pairs of orthogonal vectors $m_{1}^{\left(k\right)}$ and $m_{2}^{\left(k\right)}$ we cover the full [0, *π*) periodicity.
